# Effects of surgical and FFP2/N95 face masks on cardiopulmonary exercise capacity

**DOI:** 10.1007/s00392-020-01704-y

**Published:** 2020-07-06

**Authors:** Sven Fikenzer, T. Uhe, D. Lavall, U. Rudolph, R. Falz, M. Busse, P. Hepp, U. Laufs

**Affiliations:** 1 Klinik und Poliklinik für Kardiologie, Universitätsklinikum Leipzig, Liebigstr. 20, 04103 Leipzig, Germany; 2 Institut für Sportmedizin und Prävention, Universität Leipzig, Marschner Str. 29, 04109 Leipzig, Germany; 3 Klinik für Orthopädie, Unfallchirurgie und Plastische Chirurgie, Universitätsklinikum Leipzig, Liebigstr. 20, 04103 Leipzig, Germany

**Keywords:** Cardiopulmonary, Exercise capacity, Ventilation, Surgical masks, FFP2/N95

## Abstract

**Background:**

Due to the SARS-CoV2 pandemic, medical face masks are widely recommended for a large number of individuals and long durations. The effect of wearing a surgical and a FFP2/N95 face mask on cardiopulmonary exercise capacity has not been systematically reported.

**Methods:**

This prospective cross-over study quantitated the effects of wearing no mask (nm), a surgical mask (sm) and a FFP2/N95 mask (ffpm) in 12 healthy males (age 38.1 ± 6.2 years, BMI 24.5 ± 2.0 kg/m^2^). The 36 tests were performed in randomized order. The cardiopulmonary and metabolic responses were monitored by ergo-spirometry and impedance cardiography. Ten domains of comfort/discomfort of wearing a mask were assessed by questionnaire.

**Results:**

The pulmonary function parameters were significantly lower with mask (forced expiratory volume: 5.6 ± 1.0 vs 5.3 ± 0.8 vs 6.1 ± 1.0 l/s with sm, ffpm and nm, respectively; *p* = 0.001; peak expiratory flow: 8.7 ± 1.4 vs 7.5 ± 1.1 vs 9.7 ± 1.6 l/s; *p* < 0.001). The maximum power was 269 ± 45, 263 ± 42 and 277 ± 46 W with sm, ffpm and nm, respectively; *p* = 0.002; the ventilation was significantly reduced with both face masks (131 ± 28 vs 114 ± 23 vs 99 ± 19 l/m; *p* < 0.001). Peak blood lactate response was reduced with mask. Cardiac output was similar with and without mask. Participants reported consistent and marked discomfort wearing the masks, especially ffpm.

**Conclusion:**

Ventilation, cardiopulmonary exercise capacity and comfort are reduced by surgical masks and highly impaired by FFP2/N95 face masks in healthy individuals. These data are important for recommendations on wearing face masks at work or during physical exercise.

## Introduction

Following the outbreak of the SARS-CoV2 pandemic, use of face masks (fm) is widely recommended by international, national and local authorities [1–3]. The aim of the regulations is to reduce the respiratory droplet excretion in pre-symptomatic and asymptomatic individuals (source control). The evidence for face masks to reduce respiratory virus infections or to improve clinical outcomes is heterogeneous [4–6]. The role of fine-particle aerosols and environmental factors such as temperature and humidity on respiratory virus transmission is a matter of scientific debate [7]. However, as long as no effective treatment or vaccination against SARS-CoV2 is available, health policies need to rely on non-pharmacological interventions such as social distancing, intensified hand hygiene and the wearing of face masks. Current recommendations to wear a face mask during times of contact to other individuals affect millions of persons. Especially health care professionals are required to wear masks for long periods of time. However, the quantitative effects of medical masks on cardiopulmonary exercise capacity have never been systematically reported.

Disposable surgical masks are intended to reduce transmissions from the wearer to the patient, hand-to-face contact and facial contact with large droplets. FFP2/N95 facepiece respirators meet filtration requirements of small airborne particles, fit tightly to the wearer’s face and have been suggested to be more efficacious than surgical masks in reducing exposure to viral infections [8]. They are, therefore, widely used by health care professionals for self-protection, especially during the SARS-CoV2 pandemic. However, randomized trials did not find significant differences between FFP2/N95 and surgical masks in preventing influenza infections or respiratory illness [9, 10].

Studies on cardiopulmonary capacity have been performed using respirator masks, e.g., full facepiece masks, filtering air-purifying respirators (APR), air-supplied respirators, blower powered air-purifying respirators (PAPR), and self-contained breathing apparatus (SCBA) [11]. These respirators are better known as “gas masks*”* that are not used by health care professionals and are not suitable to be worn by the majority of the population. Data on cardiopulmonary capacity wearing medical masks are not available. Since surgical and FFP2/N95 masks are the two most widely used types of medical face masks, they were included in this study protocol.

In addition to health care professionals, information on cardiopulmonary effects of face masks in healthy adults could be important for different groups of individuals. Virus particles in respiratory droplets may be transmitted to a greater extent during different forms of physical exertion, many amateur and professional sports or activities such as singing [6, 12]. Face masks have, therefore, been discussed as means to engage in these activities for a wide range of individuals. Therefore, this randomized cross-over study aimed to provide a detailed quantification of the effect of surgical and FFP2/N95 masks on pulmonary and cardiac capacity in healthy adults.

## Materials and methods

### Subjects

The study was conducted at the Department of Cardiology, University of Leipzig. The 12 active and healthy male volunteers were recruited from medical staff. Subjects with cardiac, pulmonary or inflammatory diseases or any other medical contraindications were not included. The characteristics of the participants are shown in Table 1. The study was conducted in accordance with the latest revision of the Declaration of Helsinki and was approved by the Ethical Committee of the Medical Faculty, University of Leipzig (reference number 088/18-ek). Written informed consent was obtained from all the participants.

**Table 1:** Baseline characteristics

Parameter	Unit	Mean ± SD
Age	Years	38.1 ± 6.2
Height	cm	183 ± 7.7
Weight	kg	81.8 ± 8.4
Body mass index	kg/m^2^	24.5 ± 2.0
Sports activity	min/week	186 ± 13
Heart rate	bpm	68.1 ± 9.3
Systolic blood pressure	mmHg	126 ± 13.8
Diastolic blood pressure	mmHg	83.1 ± 6.5

*min* minute, *bpm* beats per minute

### Study design

Medical history was taken using a questionnaire. Subjects received physical examination and vital parameters, body measurements and a resting electrocardiogram (ECG). Each subject performed three incremental exertion tests (IET), one “no mask” (nm), one with surgical mask (sm) and one with FFP2/N95 mask (ffpm). The order of the masks worn was randomly assigned using the GraphPad Quickcalcs online randomization tool [13]. Tests were performed at the same time of day with a minimum of 48 h between two tests. To assess baseline respiratory function, spirometry for each setting (nm, sm, ffpm) was performed. The participants were blinded with regard to their respective test results to avoid influence by an anticipation bias. Statistical analysis was performed by an independent and fully blinded scientist who was not involved in the conduction of the tests.

### Incremental exertion test (IET)

IET were performed on a semi-recumbent ergometer (GE eBike, GE Healthcare GmbH, Solingen, Germany, Germany) at a constant speed of 60–70 revolutions per minute (rpm). The test began at a workload of 50 W with an increase of 50 W within 3 min (as a ramp) until voluntary exhaustion occurred. Each subject continued for an additional 10-min recovery period at a workload of 25 W.

### Masks

We used typical and widely used disposable FFP2/N95 protective face masks (Shaoguan Taijie Protection Technology Co., Ltd., Gao Jie, China) and surgical masks (Suavel^®^ Protec Plus, Meditrade, Kiefersfelden, Germany), both with earloops.

**Fig. 1:**
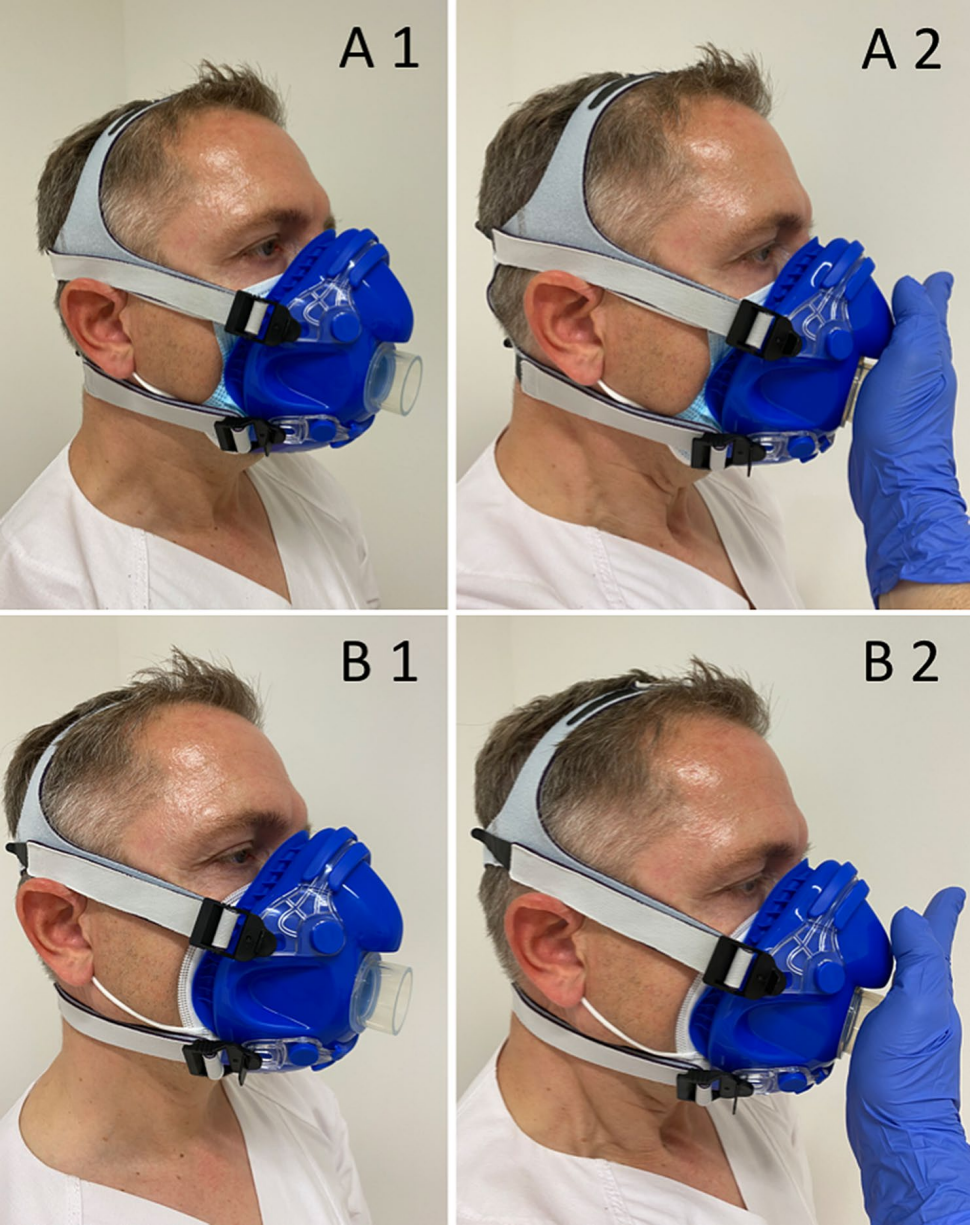
Fitting of mask and leakage test. Fitting of spirometry mask with sm (A1) and ffpm (B1) and the respective leakage tests with sm (A2) and ffpm (B2)

The spirometry mask was placed over the fm and fixed with head straps in a leak-proof manner (see Fig. 1A1, B1). After fitting the spirometry mask, subjects performed (a) inspiration and (b) expiration with maximal force. During both maneuvers, the valve of the mask was closed leading to abrupt stop of the air flow (see Fig. 1A2, B2). The fitting was carefully checked for the absence of any acoustic, sensory or visual indication of leakage (e.g., lifting of the mask, whistling or lateral airflow) by the investigators and the test person. The correct fitting and leak tightness were confirmed before each test was started.

### Measurements

Cardiac output (CO), stroke volume (SV) (measured by impedance cardiography; Physioflow, Manatec Biomedical, Macheren, France), heart rate (HR) (GE-Cardiosoft, GE Healthcare GmbH, Solingen, Germany), maximum oxygen consumption (*V*O_2max_) and minute ventilation (VE) were monitored continuously at rest, during IET and during recovery. Lung function and spirometry data were collected through a digital spirometer (Vyntus™ CPX, Vyaire Germany, Hoechberg, Germany). For each modality (nm, sm, ffpm), data of three expiratory maneuvers with 1‐min intervals were collected using the best values obtained for maximum forced vital capacity (FVC), forced expiratory volume in 1st second (FEV1), peak expiratory flow (PEF) and Tiffeneau index (TIFF). The arterio-venous oxygen difference was computed using Fick’s principle with avDO_2_ = *V*O_2_/CO. Cardiac work (CW) was measured in joules (J) and calculated according to the formula CW = SV (in m^3^) × SBP (in Pa). Capillary blood samples (55 µl) were taken from the earlobe at baseline and immediately after cessation of maximum load and analyzed (ABL90 FLEX blood gas analyzer, Radiometer GmbH, Krefeld, Germany). Blood pressures (BP) was observed at rest, every 3 min during the IET and after the first 5 min of recovery period.

### Quantification of comfort/discomfort

We used a published questionnaire published by [14] to quantify the following ten domains of comfort/discomfort of wearing a mask: humidity, heat, breathing resistance, itchiness, tightness, saltiness, feeling unfit, odor, fatigue, and overall discomfort. The participants were asked 10 min after each IET how they perceived the comfort in the test.

### Statistical analysis

All values are expressed as means and standard deviations unless otherwise stated, and the significance level was defined as *p* < 0.05. Data were analyzed using Microsoft Office Excel^®^ 2010 for Windows (Microsoft Corporation, Redmond, Washington, USA) and GraphPad Prism 8 (GraphPad Software Inc., California, USA). For distribution analysis, the D’Agostino–Pearson normality test was used. For normal distribution, comparisons were made using one-way repeated measures ANOVA with Turkey’s post hoc test for multiple comparisons. Otherwise, the Friedman non-parametric test and Dunn’s post hoc test were used. The study was powered to detect a difference of 10% in *V*O_2max_/kg between nm and ffpm.

## Results

### Pulmonary function

The results of the pulmonary function tests are shown in Table 2. Both sm and ffpm significantly reduce the dynamic lung parameters. The average reduction of FVC was −8.8 ± 6.0% with sm and −12.6 ± 6.5% with ffpm. FEV1 was −7.6 ± 5.0% lower with sm and −13.0 ± 9.0% with ffpm compared to no mask. The peak flow measurement showed that both sm and ffpm significantly reduced the PEF (−9.7 ± 11.2% and −21.3 ± 12.4%, respectively).

**Table 2:** Spirometry results

Parameter	Unit	nm	sm	ffpm	ANOVA	nm vs sm	nm vs. ffpm	sm vs ffpm
FVC	l	6.1 ± 1.0	5.6 ± 1.0	5.3 ± 0.8	**< 0.001**	**0.003**	**< 0.001**	**0.032**
FEV1	l	4.3 ± 0.7	4.0 ± 0.7	3.7 ± 0.6	**0.001**	**0.001**	**0.003**	0.068
TIFF	%	70.6 ± 9.7	71.2 ± 6.9	69.7 ± 4.9	0.635	0.934	0.900	0.520
PEF	l/s	9.7 ± 1.6	8.7 ± 1.4	7.5 ± 1.1	**< 0.001**	**0.026**	**0.001**	**0.040**

Spirometry results of health volunteers wearing no mask (nm), a surgical mask (sm) and a FFP2/N95 mask (ffpm) depicted as mean ± standard deviation

Significant results are indicated in bold

*FVC *forced vital capacity, *FEV1* forced expiratory volume in 1 s, *TIFF *Tiffenau index, *PEF* peak expiratory flow, *l* liter, *s* second

### Incremental exertion test

**Fig. 2:**
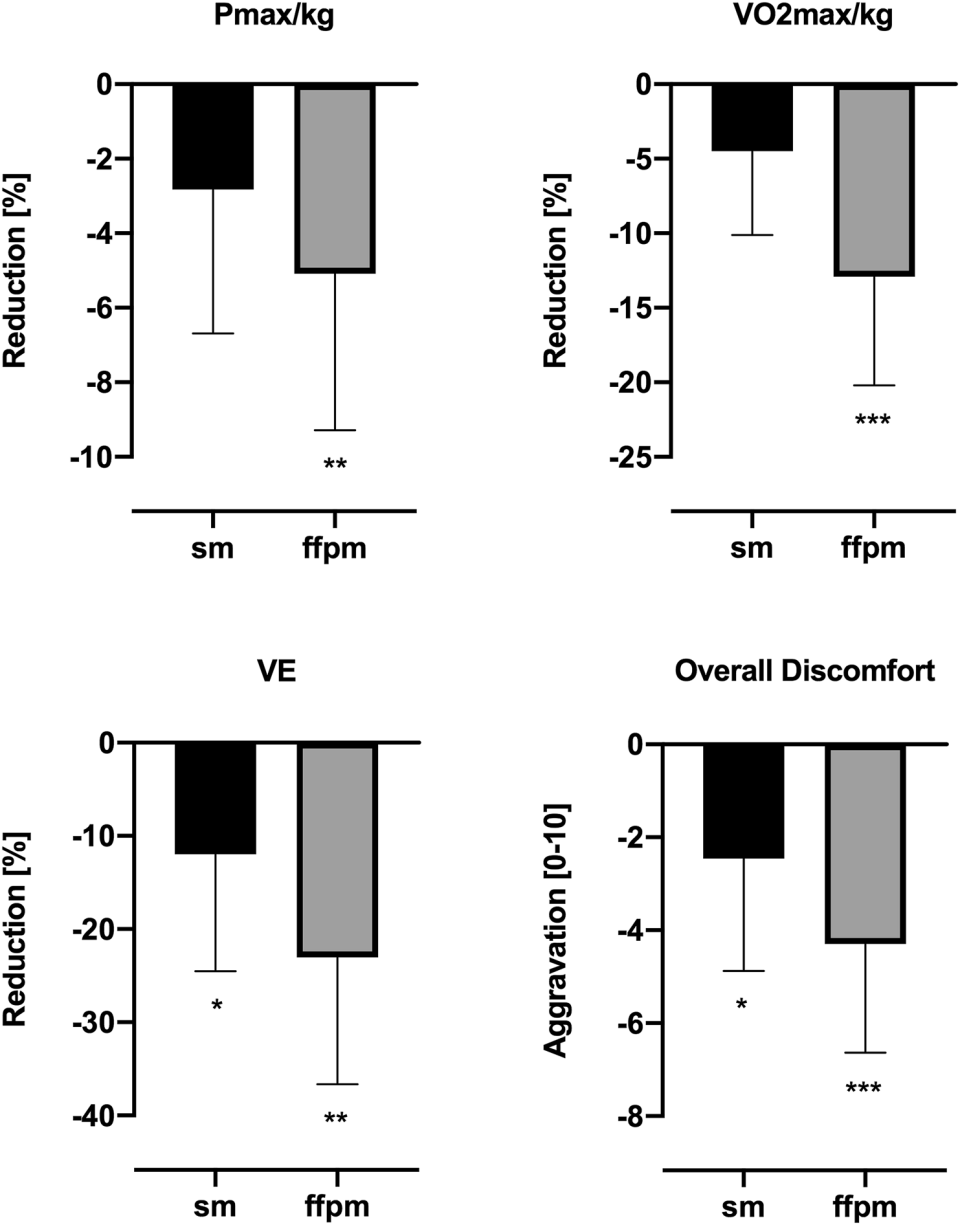
Effects of wearing a surgical mask (sm) and a FFP2/N95 mask (ffpm) compared to no mask on maximal power (*P*_max_), maximal oxygen uptake (*V*O_2max_), ventilation (VE) and overall discomfort. *p < 0.05; **p < 0.01; ***p < 0.001

The results of IET under different conditions are depicted in Table 3. None of the masks had impact on the examined parameters under resting condition. The average duration of IET compared to the test without mask was slightly decreased by −29 ± 40 s with sm (*p* = 0.07) and significantly decreased by −52 ± 45 s with ffpm (*p* = 0.005). Under maximum load, there was a large reduction of the performance measures Pmax and *V*O_2max_, especially with ffpm (Fig. 2). Furthermore, these parameters were significantly reduced in ffpm compared to sm.

**Table 3:** Results of the incremental exercise test

Incremental exertion test	Unit	nm	sm	ffpm	ANOVA	nm vs sm	nm vs. ffpm	sm vs. ffpm
Rest								
Hemodynamic parameters								
HR	bpm	66.2 ± 9.3	66.2 ± 11.8	66.2 ± 7.2	1.000	1.000	1.000	1.000
SV	ml	100 ± 17.7	105 ± 22.3	103 ± 21.0	0.280	0.354	0.310	0.863
CO	l/min	6.3 ± 0.7	6.6 ± 0.7	6.6 ± 0.9	0.314	0.542	0.248	0.985
avDO_2_	%	5.4 ± 1.5	4.7 ± 1.3	5.1 ± 0.9	0.346	0.307	0.837	0.623
SBP	mmHg	117 ± 8.7	122 ± 12.3	121 ± 12.0	0.399	0.474	0.529	0.977
DBP	mmHg	81.9 ± 6.1	80.1 ± 6.6	81.0 ± 6.2	0.569	0.494	0.836	0.907
Pulmonary parameters								
VE	l/min	10.5 ± 2.5	10.3 ± 2.6	10.4 ± 1.9	0.822	0.898	0.967	0.958
Breathing frequency	brpm	14.8 ± 2.2	12.9 ± 2.9	12.5 ± 2.7	**0.006**	0.051	**0.016**	0.601
VT	l	0.7 ± 0.2	0.8 ± 0.2	0.9 ± 0.2	0.146	0.465	0.125	0.770
Metabolic parameters								
pH		7.41 ± 0.02	7.44 ± 0.06	7.42 ± 0.02	0.166	0.278	0.558	0.422
PCO_2_	mmHg	40.2 ± 3.4	39.3 ± 3.6	39.3 ± 2.2	0.094	0.179	0.213	0.998
PO_2_	mmHg	111 ± 4.3	117 ± 23.1	122 ± 22.1	0.465	0.824	0.487	0.787
Lactate	mmol/l	1.00 ± 0.27	0.78 ± 0.26	1.04 ± 0.52	0.125	**0.003**	0.962	0.281
Maximum load								
Peformance								
* P*_max_	W	277 ± 45.9	269 ± 45.1	263 ± 41.7	**0.002**	0.071	**0.005**	**0.018**
* P*_max_/kg	W/kg	3.40 ± 0.5	3.30 ± 0.5	3.22 ± 0.4	**0.001**	0.066	**0.005**	**0.019**
* V*O_2max_/kg	(ml/min)/kg	39.7 ± 5.8	37.9 ± 6.0	34.5 ± 5.3	**< 0.001**	0.063	**0.001**	**0.013**
Hemodynamic parameters								
HR	bpm	187 ± 8.3	183 ± 9.2	182 ± 11.2	0.106	**0.031**	0.107	0.964
SV	ml	151 ± 26.4	165 ± 35.0	164 ± 20.4	0.086	0.166	0.074	0.979
CO	l/min	25.8 ± 4.2	27.3 ± 5.6	27.0 ± 3.8	0.342	0.435	0.422	0.964
avDO_2_	%	12.8 ± 2.8	11.5 ± 2.2	10.5 ± 2.0	**0.002**	0.084	**0.007**	0.172
SBP	mmHg	214 ± 18.2	212 ± 28.5	210 ± 18.8	0.901	0.984	0.905	0.954
DBP	mmHg	88.8 ± 9.6	95.8 ± 36.7	89.8 ± 8.8	0.582	0.779	0.959	0.847
Pulmonary parameters								
VE	l/min	131 ± 27.8	114 ± 23.3	98.8 ± 18.6	**0.001**	0.048	**0.003**	0.009
Breathing frequency	brpm	40.9 ± 5.1	39.3 ± 6.2	36.8 ± 5.9	**0.019**	0.518	**0.024**	0.138
VT	l	3.2 ± 0.7	2.9 ± 0.5	2.7 ± 0.4	**0.016**	0.255	**0.021**	0.102
Metabolic parameters								
pH		7.27 ± 0.05	7.32 ± 0.10	7.31 ± 0.06	0.158	0.216	0.065	0.989
PCO_2_	mmHg	34.2 ± 3.8	34.3 ± 5.9	34.9 ± .0	0.726	0.999	0.560	0.943
PO_2_	mmHg	107 ± 20.5	116 ± 23.7	116 ± 23.2	0.502	0.714	0.339	0.996
Lactate	mmol/l	12.8 ± 3.09	11.0 ± 3.91	10.8 ± 3.12	**0.049**	0.132	0.105	0.985
Recovery								
Hemodynamic parameters								
HRR-1 min	bpm	−39.7 ± 15.9	−38.1 ± 9.2	−39.9 ± 11.2	0.203	0.055	0.611	0.781
HRR-5 min	bpm	−72.5 ± 24.1	−77.6 ± 11.5	−77.3 ± 10.9	0.874	0.938	0.855	0.991

Results of the incremental exercise test of health volunteers wearing no mask (nm), a surgical mask (sm) and a FFP2/N95 mask (ffpm) depicted as mean ± standard deviation

Significant results are indicated in bold

*HR* heart rate, *P* power, *SV* stroke volume, *CO* cardiac output, *avDO*_*2*_ arterio-venous oxygen content difference, *SBP* systolic blood pressure, *DBP* diastolic blood pressure, *V*O_2_ oxygen uptake, *VE* ventilation, *VT* tidal volume, *PCO*_*2*_ partial pressure of carbon dioxide, *PO*_*2*_ partial pressure of oxygen, *HRR* heart rate recovery, *bpm* beats per minute, *W* Watt, *brpm* breaths per minute

Assessment of the hemodynamic parameters (Table 3) showed that ffpm decreased avDO_2_ by 16.7 ± 11.2% compared to nm. Stroke volume and cardiac output and cardiac work did not differ significantly (nm: 4.3 ± 0.8 J, sm: 4.7 ± 1.4 J, ffpm: 4.6 ± 0.9 J; *p* = 0.29).

The masks showed a marked effect on pulmonary parameters: VE for both sm and ffpm was significantly reduced by −12.0 ± 12.6% and −23.1 ± 13.6%, respectively, compared to nm (see Table 3; Fig. 1). Compared to nm, tests with ffpm showed a significant reduction in breathing frequency with an additional decrease in tidal volume (−9.9 ± 11.3% and −14.4 ± 13.0%, respectively). At the same time, a longer inhalation time was observed (sm: 12 ± 15%, *p* = 0.043; ffpm: 19 ± 16%, *p* = 0.005). There were no differences in exhalation time.

Measurements of the metabolic parameters pH, PCO_2_, PO_2_ and lactate and the heart rate recovery did not differ significantly between the three tests (Table 3).

### Perceived discomfort

Subjective ratings for different sensations and overall discomfort for sm and ffpm compared to nm are depicted in Table 4. In general, the negative ratings for all items of discomfort increased consistently and significantly from sm to ffpm. There were several-fold negative reports for the ffpm compared to nm and sm for breathing resistance. The relative aggravation in overall discomfort compared to the standard procedure for spiroergometric tests is shown in Fig. 2.

**Table 4:** Perceived discomfort

Discomfort	nm	sm	ffpm	ANOVA	nm vs sm	nm vs ffpm	sm vs ffpm
Humid	2.4 ± 2.0	4.9 ± 3.2	5.9 ± 2.2	**0.003**	0.069	**0.001**	0.402
Hot	2.0 ± 1.3	4.2 ± 2.4	6.2 ± 2.3	**< 0.001**	**0.012**	**< 0.001**	**0.024**
Breath resistance	1.7 ± 1.2	5.4 ± 1.9	7.4 ± 2.5	**< 0.001**	**0.001**	**< 0.001**	**0.045**
Itchy	1.1 ± 1.0	3.4 ± 3.1	4.9 ± 2.6	**0.002**	**0.030**	**0.001**	0.331
Tight	1.9 ± 1.8	3.9 ± 2.6	6.5 ± 2.3	**< 0.001**	**0.035**	**< 0.001**	**0.021**
Salty	0.7 ± 1.1	1.6 ± 1.5	3.5 ± 2.8	**0.003**	0.261	**0.012**	**0.023**
Unfit	1.4 ± 1.2	3.3 ± 2.3	5.4 ± 2.3	**< 0.001**	**0.009**	**< 0.001**	**0.016**
Odor	1.4 ± 2.2	1.2 ± 0.9	3.6 ± 2.8	**0.011**	0.956	0.056	**0.036**
Fatigue	2.7 ± 2.2	5.8 ± 2.5	6.5 ± 2.6	**< 0.001**	**0.002**	**0.001**	0.394
Overall discomfort	2.8 ± 2.2	5.2 ± 2.1	7.0 ± 1.7	**< 0.001**	**0.012**	**< 0.001**	**0.005**

Results of the questionnaire [14] quantitating ten domains of comfort/discomfort of wearing a surgical mask (sm) and a FFP2/N95 mask (ffpm) compared to no mask on a scale from 0 (no discomfort at all) to 10 (maximal discomfort) depicted as mean ± standard deviation

Significant results are indicated in bold

## Discussion

This first randomized cross-over study assessing the effects of surgical masks and FFP2/N95 masks on cardiopulmonary exercise capacity yields clear results. Both masks have a marked negative impact on exercise parameters such as maximum power output (*P*_max_) and the maximum oxygen uptake (*V*O_2max_/kg). FFP2/N95 masks show consistently more pronounced negative effects compared to surgical masks. Both masks significantly reduce pulmonary parameters at rest (FVC, FEV1, PEF) and at maximum load (VE, BF, TV). Furthermore, wearing the masks was perceived as very uncomfortable with a marked effect on subjective breathing resistance with the FFP2/N95 mask.

### Pulmonary function

Spirometry showed reduced FVC, FEV1 and PEF with the surgical mask and even greater impairments with the FFP2/N95 mask. Wearing the FFP2/N95 mask resulted in a reduction of *V*O_2max_ by 13% and of ventilation by 23%. These changes are consistent with an increased airway resistance [15]. Studies testing increased upper airway obstruction induced by added resistance at the mouth report similar effects on the lung functions parameter with increased breathing resistance [16]. The reduction in ventilation resulted from a lower breathing frequency with corresponding changes of the inhaling and exhaling time and a reduced tidal volume. This is in agreement with the effects of respiratory protective devices or additional external breathing resistance [16, 17]. The increased breathing resistance, which is likely higher during stress, leads to an elevated breathing work and a limitation of the ventilation. The data of this study are obtained in healthy young volunteers, the impairment is likely to be significantly greater, e.g., in patients with obstructive pulmonary diseases [18]. From our data, we conclude that wearing a medical face mask has a significant impact on pulmonary parameters both at rest and during maximal exercise in healthy adults.

### Cardiac function

Increased breathing resistance in ffpm and sm requires more work of the respiratory muscles compared to nm leading to higher oxygen consumption. Additionally, a significant proportion of cardiac output is directed via different mechanisms, e.g., sympathetically induced vasoconstriction, to the respiratory musculature [19]. Furthermore, the increased breathing resistance may augment and prolong inspiratory activity leading to more negative intrathoracic pressure (ITP) for longer durations. This assumption is supported by the findings on inspiration times which were higher while wearing a fm. Prolonged and more negative ITP increases the cardiac preload and may lead to higher SV at the one hand which is consistent with our results showing a statistical trend towards higher SV while wearing ffpm or sm [20, 21]. In addition, cardiac afterload increases because of an increased transmural left-ventricular pressure resulting in enhanced myocardial oxygen consumption [22]. In these healthy volunteers, functional cardiac parameters do not differ significantly at baseline, at maximal load and during recovery. However, there is a non-significant trend towards a higher cardiac work (Joule) compared to the test without mask. This is of relevance since significantly less watts (−5%) was achieved in the tests with masks. The relation of cardiac power to the total power is approximately 10% lower with ffpm. These data suggest a myocardial compensation for the pulmonary limitation in the healthy volunteers. In patients with impaired myocardial function, this compensation may not be possible.

### Performance

The measurements show that surgical masks, and to a greater extent FFP2/N95 masks, reduce the maximum power. *P*_max_ (Watt) depends on energy consumption and the maximum oxygen uptake (*V*O_2max_). The effect of the masks was most pronounced on *V*O_2max_. Since the cardiac output was similar between the conditions, the reduction of *P*_max_ was primarily driven by the observed reduction of the arterio-venous oxygen content (avDO_2_). Therefore, the primary effect of the face masks on physical performance in healthy individuals is driven by the reduction of pulmonary function. In addition, the auxiliary breathing muscles have been described to induce an additional afferent drive which can contribute to an increase of the fatigue effect [23–25].

The performance of several different populations may be significantly affected by face masks. For athletes the use of fm will reduce physical performance. Less pronounced but mechanistically similar effects have been observed for mouthguards [26–28]. The increased breathing resistance is especially problematic for patients with chronic obstructive pulmonary diseases. Patients with diffusion disorders have reduced capacity to compensate due to the reduced tidal volume. Another example of a population at risk is patients with heart failure. The observed mechanisms may lead to more severe symptoms in individuals with impaired capacity for myocardial compensation.

### Discomfort

Health care professionals and others are faced with significant psychological distress during viral outbreaks [29]. Measures to maintain the quality of life both during emergency situations and long term care are increasingly important. Adequate personal protective equipment and adequate rest are considered keys to reduce the risk of adverse psychological outcomes [29]. Our sample primarily consisted of physicians working at a university hospital who are very familiar with medical masks and have a positive attitude towards personal protection. Our data show that FM leads to severe subjective discomfort during exercise. FFP2/N95 masks are perceived as more uncomfortable than sm. In particular, breathing resistance, heat, tightness and overall discomfort are the items with the greatest influence on subjective perception. This finding is in agreement with the literature [14, 30]. Wearing of fm is perceived as subjectively disturbing and is accompanied by an increased perception of exertion. It is likely that the masks negatively impact on the dynamics of perception especially at the limit of exercise tolerance [31, 32]. In addition to the severe impact on ventilation, the data suggest the associated discomfort as a second important reason for the observed impairment of physical performance.

## Limitations of the study

The sample consisted of relatively young, healthy, male participants. The data cannot be extrapolated to other populations but set the stage to assess the effects of the face masks in elderly and in patients with pulmonary and with cardiac diseases. This study is the largest cross-over study to date comparing acute cardiopulmonary effects with and without common face masks, however, independent repetition and larger sample size is always welcome. The external validity concerning surgical masks (relevant leakage to eyes and ears in daily life) may be reduced because of the laboratory conditions where the sm was completely sealed by the spirometry mask. Cardiac parameters obtained by impedance cardiography may be overestimated using absolute values [33]. However, thoracic impedance cardiography is well established for the quantification of intra-individual changes in SV and CO [34–36].

## Conclusion

Medical face masks have a marked negative impact on cardiopulmonary capacity that significantly impairs strenuous physical and occupational activities. In addition, medical masks significantly impair the quality of life of their wearer. These effects have to be considered versus the potential protective effects of face masks on viral transmissions. The quantitative data of this study may, therefore, inform medical recommendations and policy makers.
